# Comparing Apples and Oranges: Fold-Change Detection of Multiple Simultaneous Inputs

**DOI:** 10.1371/journal.pone.0057455

**Published:** 2013-03-04

**Authors:** Yuval Hart, Avraham E. Mayo, Oren Shoval, Uri Alon

**Affiliations:** Department of Molecular Cell biology, Weizmann Institute of Science, Rehovot, Israel; Institute for Systems Biology, United States of America

## Abstract

Sensory systems often detect multiple types of inputs. For example, a receptor in a cell-signaling system often binds multiple kinds of ligands, and sensory neurons can respond to different types of stimuli. How do sensory systems compare these different kinds of signals? Here, we consider this question in a class of sensory systems – including bacterial chemotaxis- which have a property known as fold-change detection: their output dynamics, including amplitude and response time, depends only on the relative changes in signal, rather than absolute changes, over a range of several decades of signal. We analyze how fold-change detection systems respond to *multiple* signals, using mathematical models. Suppose that a step of fold F_1_ is made in input 1, together with a step of F_2_ in input 2. What total response does the system provide? We show that when both input signals impact the same receptor with equal number of binding sites, the integrated response is multiplicative: the response dynamics depend only on the product of the two fold changes, F_1_F_2_. When the inputs bind the same receptor with different number of sites n_1_ and n_2_, the dynamics depend on a product of power laws, 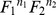. Thus, two input signals which vary over time in an inverse way can lead to no response. When the two inputs affect two different receptors, other types of integration may be found and generally the system is not constrained to respond according to the product of the fold-change of each signal. These predictions can be readily tested experimentally, by providing cells with two simultaneously varying input signals. The present study suggests how cells can compare apples and oranges, namely by comparing each to its own background level, and then multiplying these two fold-changes.

## Introduction

Sensory systems can often detect multiple types of inputs. For example, receptors in cells often bind multiple ligands. Well-known examples are bacterial chemotaxis receptors where each receptor detects several chemo-attractants; Tar, for example, can bind the attractants aspartate and maltose, and the repellents Ni and Co ions [1]–[3]. In mammalian cells, the EGF receptor can bind a family of hormone ligands including EGF, TGF-α, EPR, amphiregulin and more [4], [5]. Similar multi-input situations occur in neuronal sensory systems: Sensory neurons in *C. elegans* can detect multiple inputs, as exemplified by the neuron ASH which can detect both touch and noxious chemicals [6], [7]. In addition to detection of multiple inputs by the same detector component (receptor or neuron), often multiple detectors impinge on the same downstream integration unit to produce an output.

Thus, a question of general interest is how sensory systems interpret multiple input signals, each of which can change over time.

We address this question for sensory systems which have fold-change detection, a property recently defined in theoretical and experimental studies [8]–[15]. Fold-change detection means that the system responds to fold (relative) changes in the input rather than the input’s absolute levels – at least over a range of several decades of input signal strength. Thus, a step change in input levels from 1 to 2 and a step from 2 to 4 results in precisely the same response dynamics, including amplitude and duration. The same output dynamics occur because both steps have the same fold change, F = 2. Fold change detection (FCD) goes beyond Weber’s law [16], [17], a feature commonly found in physiological sensory systems. Weber’s law states that response amplitude is proportional to the relative change in signal; in FCD systems, the entire response profile including both its amplitude and its duration (adaptation time) is a function only of fold changes. Recent experiments on bacterial chemotaxis have demonstrated FCD over a 3-order of magnitude range of the chemo-attractant alpha-methyl aspartate and also glucose [12], [13].

Here we ask how fold-change detection systems respond to *multiple* signals. Suppose that a step of F_1_ is made in input 1, together with a step of F_2_ in input 2: what total response does the system provide? We show that when both independent signals impact the same receptor with equal number of binding sites, the integrated response is multiplicative: the response dynamics depend only on the product of the two fold changes, F_1_F_2_.

In cases where the inputs bind the same receptor with different number of sites, the dynamics depend only on a product of power laws, resulting in a log-linear formula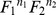. In this case, a step of F_1_ in input 1 together with a step F_2_ in input 2 is equivalent to a step of 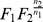 (or 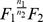) in ligand one (two) where ligand two (one) is kept constant.

When the two inputs affect different receptors, other types of integration may be found and generally the system is not constrained to respond in a way determined by the product of the fold change of each signal.

Finally, we analyze the constraints of signal integration in a general three layered sensory system with FCD. We find a broad set of systems that integrate signals in a log-linear form similar to the case of shared receptors.

## Results

### Fold-change Detection Mechanism in the Chemotaxis System of *E.coli*, the Case of a Single Input

**Figure 1:**
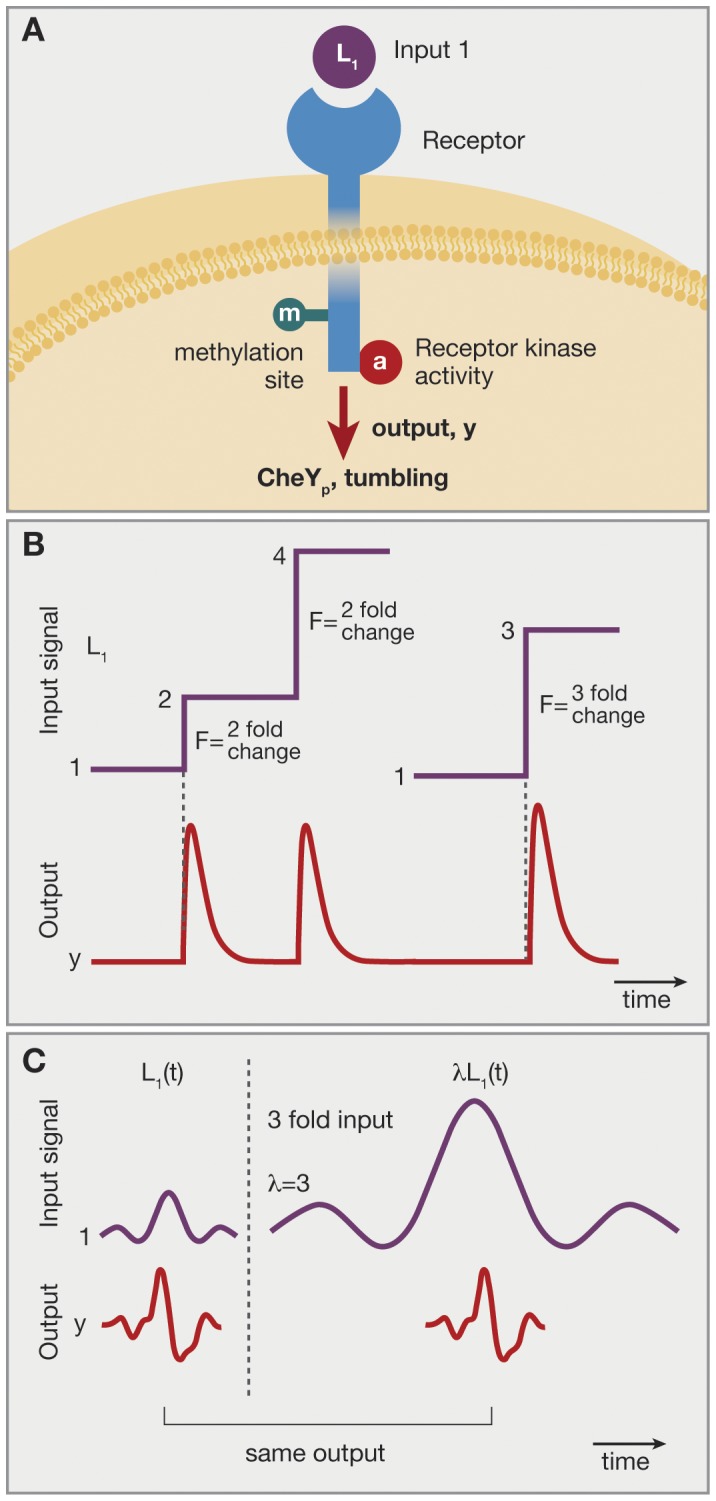
Fold change detection (FCD) in bacterial chemotaxis in the single input situation and its experimental test by Lazova et al. (A) The chemoreceptor Tar binds the ligand MeAsp (L_1_). Its associated kinase activity is modified by methylation. The output is CheYp which lead to bacterial tumbles. (B) Analysis of Shoval et al of the Tu et al model of chemotaxis predicted that the system shows fold change detection. For example, two steps of the same fold of L_1_ should yield the same output dynamics, including the same amplitude and response time, over a wide range of background L_1_ concentrations. (C) Lazova et al tested this prediction by means of *E.coli* strains in which the output (CheYp level) is read out optically – by means of FRET - in a flow cell in which L_1_ levels can be changed over time. Over a 2-order of magnitude range of concentrations, output was invariant to multiplying L_1_(t) by a constant factor. Data from Lazova et al [12].

For completeness, and to provide nomenclature for the following results, we first summarize the results of Ref [9] on the fold change detection (FCD) mechanism in the chemotaxis system of *E.coli*. The dynamic equations are based on the model of Tu, Shimizu and Berg [18] which describes the chemotaxis response to temporal signals in good agreement with a wide range of experiments [19]–[23]. In the model, the variables are the methylation level of the receptors, and the activity of the receptor-kinase complex *a* (see Fig.1A). Briefly, chemoattractant binding lowers receptor methylation, which lowers kinase activity, leading the cells to reduce their rate of random direction changes (called tumbles) and thus swim on average up the gradient of attractant [2], [24].

The model equations describe the change in methylation, m, due to an integral feedback loop [25]–[28], which provides exact adaptation to the activity *a* at a steady state value a_0_:
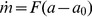
(1)where 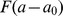 is a decreasing function that crosses zero when a = a_0_ (which makes a_0_ the fixed point of the system’s activity).

Receptor activity in the Tu et al model is described using a Monod-Wyman-Changeux (MWC) mechanism [29] for a receptor cluster with n binding sites which is affected both by ligand binding and methylation:
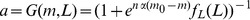
(2)


Here, 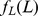 is the receptor activity dependence on input ligand levels L, given by a standard MWC term [18], [30]:
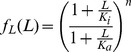
(3)


Where 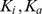 are the receptor affinities of the inactive and active states for the ligand L. This results in a power law form for the activity - 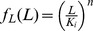
- in the region 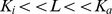.

The power-law region spans several orders of magnitude of concentration. For example, the Tar receptor and the attractant methyl-aspartate show a power-law region which spans a 160 fold range in ligand levels [30], between 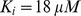
and 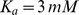.

By using the transformation 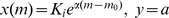 (where y is the receptor activity and x represents an effective methylation-dependent affinity) one finds a set of dynamic equations in the above mentioned concentration range (1).
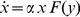
(4)

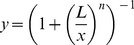
(5)


These equations show FCD, because they obey the sufficient conditions for FCD presented by Shoval et al. [9], [11]: the system is stable [31] and the equation for y(t) (Eq. (5)) remains invariant under the transformation 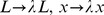 for 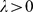.

Thus, the output y(t) for a given input stimulus 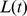 is equal to the dynamics for the same input multiplied by a constant, 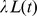. Intuitively, FCD can be seen by comparing two step stimuli with equal fold-change. The response of a step from input level 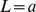 to 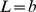, has exactly the same dynamics - response amplitude and adaptation time - as a step from level 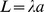 to 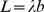. Both of these steps have the same fold change F = b/a.

The FCD property applies as long as the inputs are within the range of concentrations 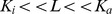. Note especially that stimulations starting from L = 0, including the pioneering chemotaxis experiments of Berg and Tedesco [19] in which response time was found to be additive in input ligand levels, are outside the FCD range.

Recent experiments by Lazova et al. [12] tested the prediction of FCD in *E coli* chemotaxis. Cells in which chemotaxis activity can be read out fluorescently [20], were placed in a flow chamber allowing control of input stimuli 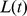. Cells were presented with stimuli 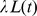 of alpha-methyl-aspartate with different values of 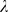. The experiments revealed two FCD regimes in which the output is independent of 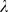
(see Fig.1C). These regimes together span three orders of magnitude of ligand concentrations. In another set of experiments, Masson et al [13] used a noninvasive imaging method in a microfluidic device to measure the chemotactic response function of E. coli. They find that both for alpha-methyl-aspartate and glucose, the response shape does not depend on ligand background levels over a range of at least 3 orders of magnitude, implying FCD.

In the following, we expand the model to two different input ligands binding the same receptor.

### Two Inputs for the Same Receptor with an Equal Number of Binding Sites are Integrated as the Product of their Fold Changes

We start by considering a MWC model of a receptor which binds two different input ligands in an independent way [3]. We wish to characterize the response to fold changes in the two ligand concentrations, F_1_ and F_2_ respectively, in terms of the equivalent fold change needed in one of the ligands. We define E_1_(F_1_,F_2_) as the fold change in input ligand one (L_1_), keeping ligand two (L_2_) constant, which produces the same output dynamic y(t) as a simultaneous fold change of F_1_ in ligand one (L_1_) and F_2_ in ligand two (L_2_). An equivalent definition applies for E_2_(F_1_,F_2_). In this section we show that for the case of equal number of binding sites for both ligands, 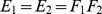.

**Figure 2:**
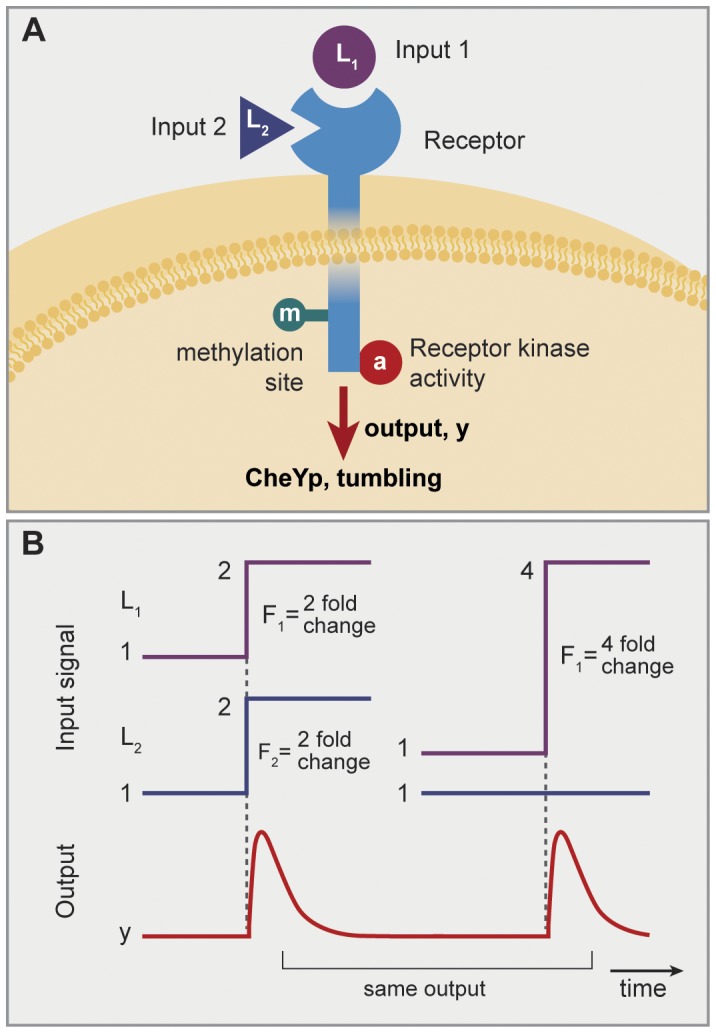
Product-rule integration of two input signals in an FCD system, where both inputs bind the same receptor with the same number of sites. (A) Example of a receptor that binds to input ligands, the Tar receptor that binds both MeAsp, and maltose. (B) Response to simultaneous steps of L_1_ and L_2_ of folds F_1_ and F_2_ is dependent only on F_1_F_2_. Thus, steps with F_1_ = 4 and F_2_ = 1 shows the same output dynamics as two steps with F_1_ = 2 and F_2_ = 2, since in both cases F_1_F_2_ = 4. This holds as long as all concentrations are not too high or too low, that is - only in the FCD concentration range.



Consider a MWC model for receptor activity dependent on two input ligands, following ref [3], [9]. Each ligand has n binding sites and binding is independent of the other ligand (see Fig.2A). The resulting activity dependence on the input ligands L_1_ and L_2_ is (as shown in Eq. 7, 8 of ref [3]):

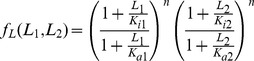
(6)and 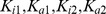 are the receptor’s affinities in the inactive and active states for the ligands L_1_ and L_2_ respectively. Similarly to the single ligand case, in the range where 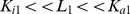 and 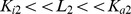, the activity dependence on both ligand levels is a power law,



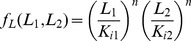
(7)Repeating the analysis of the previous section, we define the transformation 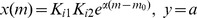 to find the following set of equations
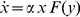
(8)

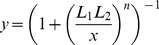
(9)where we used the activity function calculated in Eq.(7). This set of equations again obeys the FCD conditions of Shoval et al [9]. Note that the effective input in the equations is the product 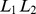. The system is therefore FCD for 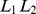. In particular, a simultaneous step of fold F_1_ in L_1_ and fold F_2_ in L_2_ yields the same response as a step of size F_1_F_2_ in ligand L_1_ alone, keeping L_2_ constant (and equivalently a step of F_1_F_2_ in L_2_ keeping L_1_ constant, see Fig. 2B). Thus, 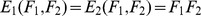.

**Figure 3:**
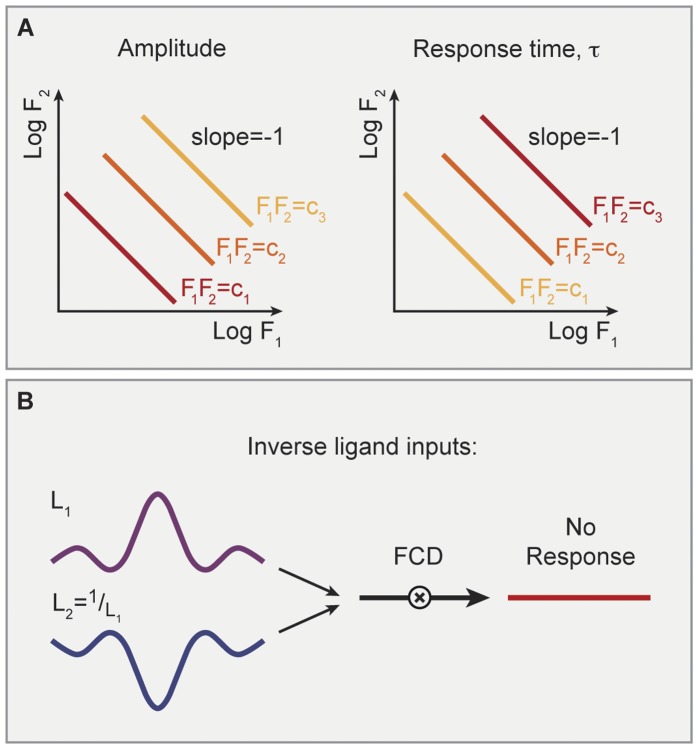
Log linear integration of signals. (A) Two inputs to the same receptor with equal number of binding sites are predicted to show linear equi-amplitude and equi-response time curves in the (log F_1_, log F_2_) plane. Results shown are for the model of bacterial chemotaxis, with parameters y_0_ = 1,n = 1. With increasing values of F_1_F_2_ - amplitude rises (red to yellow) and response time decreases (yellow to red) (B) Two simultaneous input time curves which are inverse, namely L_1_(t) = c/L_2_(t), are predicted to yield no response.

To test this product-rule for interpretation of multiple signals, consider the following potential experiment. One measures the response (amplitude and pulse duration) for many combinations of simultaneous step changes of fold F_1_ in ligand one (L_1_) and fold F_2_ in ligand two (L_2_). Plotting the amplitude or duration in the plane whose coordinates are log F_1_ and log F_2_, one should obtain straight line contours with slope minus one (see Fig.3A). An interesting implication of the multiplicative nature of the signal integration is that two signals which are inverse to each other, 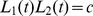, should yield no response (see Fig. 3B).

It should be noted that the range of inputs over which the product rule applies is finite, namely, 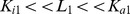 and 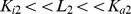. As mentioned above, this range is about 160-fold for the Tar receptor and its ligand methyl-aspartate.

In Material S1 we present results for the cases when ligand bindings are not independent (interacting or exclusive). We show that for a certain range of concentrations and parameters FCD still holds when ligand binding are interacting, but cannot be attained when ligand binding is strictly mutually exclusive.

We next treat the case where the number of binding sites differs between the two ligands.

### Two Inputs for the Same Receptor with Differing Number of Binding Sites are Integrated in a Log-linear Manner

We now consider the case where the number of binding sites for each ligand is not equal. This may relate, for example, to models of heterogeneous bacterial chemoreceptor complexes [3]. We denote the number of binding sites for ligand one by n_1_ and for ligand two by n_2_. When ligand levels are in the range where fold change detection holds for each ligand alone (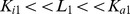 and 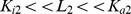) Eq. (7) takes the following form:
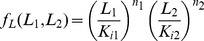
(10)


This form suggests that the two input signals are weighted differently. One can write Eq. (10) as:
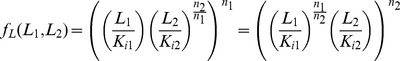
(11)


**Figure 4:**
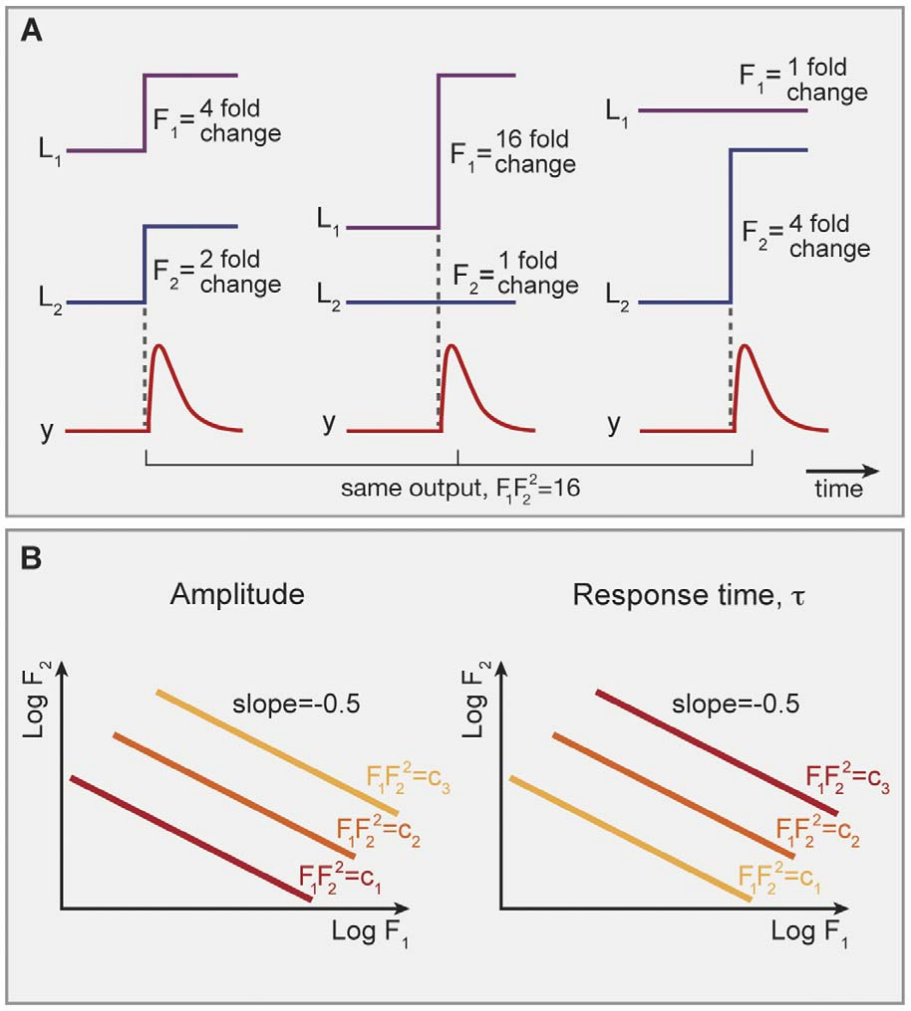
Power-law product rule for integration of two input signals in an FCD system, where both inputs bind the same receptor with different number of sites. (A) Let L_1_ have one binding site, and L_2_ have two sites. Response to simultaneous steps of L_1_ and L_2_ of folds F_1_ and F_2_ is dependent only on 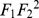. Thus, steps with F_1_ = 4 and F_2_ = 2 shows the same output dynamics as two steps with F_1_ = 16 and F_2_ = 1 or two steps of F_1_ = 1 and F_2_ = 4, since in all cases 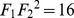
(B) Linear equi-response contours in the (log F_1_, log F_2_) plane. With increasing values of 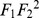
- amplitude rises (red to yellow) and response time decreases (yellow to red).

Thus, the integration of two ligands is not equivalent to the product of their fold changes. Instead, the dynamic response for a simultaneous fold change of F_1_ and F_2_ in the two inputs results in equivalent dynamics as a fold change of 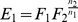 in ligand one (L_1_), keeping ligand two (L_2_) constant. Similarly, for the second ligand 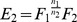 (see Fig.4A). Again, experimentally mapping the response for different fold change steps of size F_1_ and F_2_, should yield straight line contours in the (logF_1_, logF_2_) plane, with slopes of 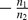
(See Fig. 4B).

We next describe a generalization of these results for a system with a shared internal layer of inputs integration. This generalization suggests that such systems have an invariant dynamic response property of the two folds.

### A General FCD System with a Single Internal Layer Component Integrates Inputs in a Log-linear Manner

We next describe signal integration for a general three layer sensing system composed of a layer of two inputs (u_1_,u_2_), an internal layer with one degree of freedom (denoted x, e.g. the modification state of a receptor binding two ligands) and an output (denoted y). This sensing system is assumed to be a FCD system for each input, and the input levels are assumed to be in the FCD regime, so that the system responds to fold changes in either input. The model allows for any type of interaction between the three computational layers, including activation and inhibition, feed-forward and feedback. The dynamic equations for x and y are generally.
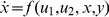
(12)

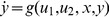
(13)


For the system to obey FCD, the above set of equations must obey scalar symmetry [9], [11]: be invariant to multiplications by a scalar in u_1_ and u_2_, 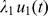 and 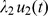. Consider the following transformation of the inputs of the system:
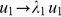
(14)

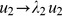
(15)

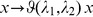
(16)


In the methods section, we analyze the more general case in which 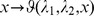.

For the system to be FCD in both inputs, the dynamics of y must remain the same. Therefore we have the condition: 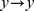. These considerations yield the following transformed set of dynamic equations:
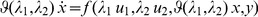
(17)

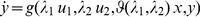
(18)


This results in the following equality between the two dynamic sets of equations:
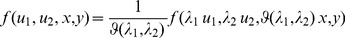
(19)

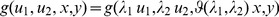
(20)


Following Euler’s homogeneity rule, one differentiates Eqn. (19–20) with respect to 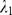 and 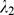, at the point 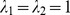 and gets the following constraints on the functional form of the functions *f* and *g*:
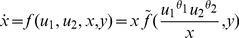
(21)

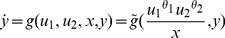
(22)where 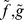 are the scaled dynamic functions and the power laws are determined by the partial derivative of the transformation function 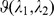
with respect to 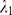 and 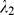 at the point 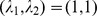 as follows: 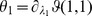 and 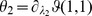. Therefore, the only allowed form of input integration is one where the two signals are integrated as the product of power-laws of the signals:



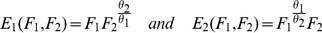
(23)Thus, log E_1_ is a linear combination of log F_1_ and log F_2_. For example, the transformation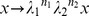, yields the conserved invariant of the dynamic response, 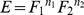 which means that the equivalent response in each signal is 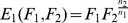 and 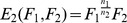. A solution for the general case of two input signals with one shared internal layer is presented in the Methods section.

### A FCD System with Two Internal Layer Components does not Necessarily Integrate Inputs in a Log-linear Manner

We next show that a system with a separate internal component for each input (e.g. two different receptors for two ligands) does not necessarily yield an invariant dynamic response.

Consider the case where each input signal has its own internal layer component, x_1_ and x_2_. The general dynamic equations are
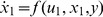
(24)

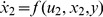
(25)

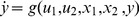
(26)


Following the same procedure of the previous section yields these constraints on the differentials functional form:
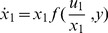
(27)

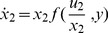
(28)

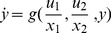
(29)


Therefore, each signal is scaled by its own internal layer component (x_i_), and there are no constraints on their integration since the two internal components can decouple the effects of each signal.

**Figure 5:**
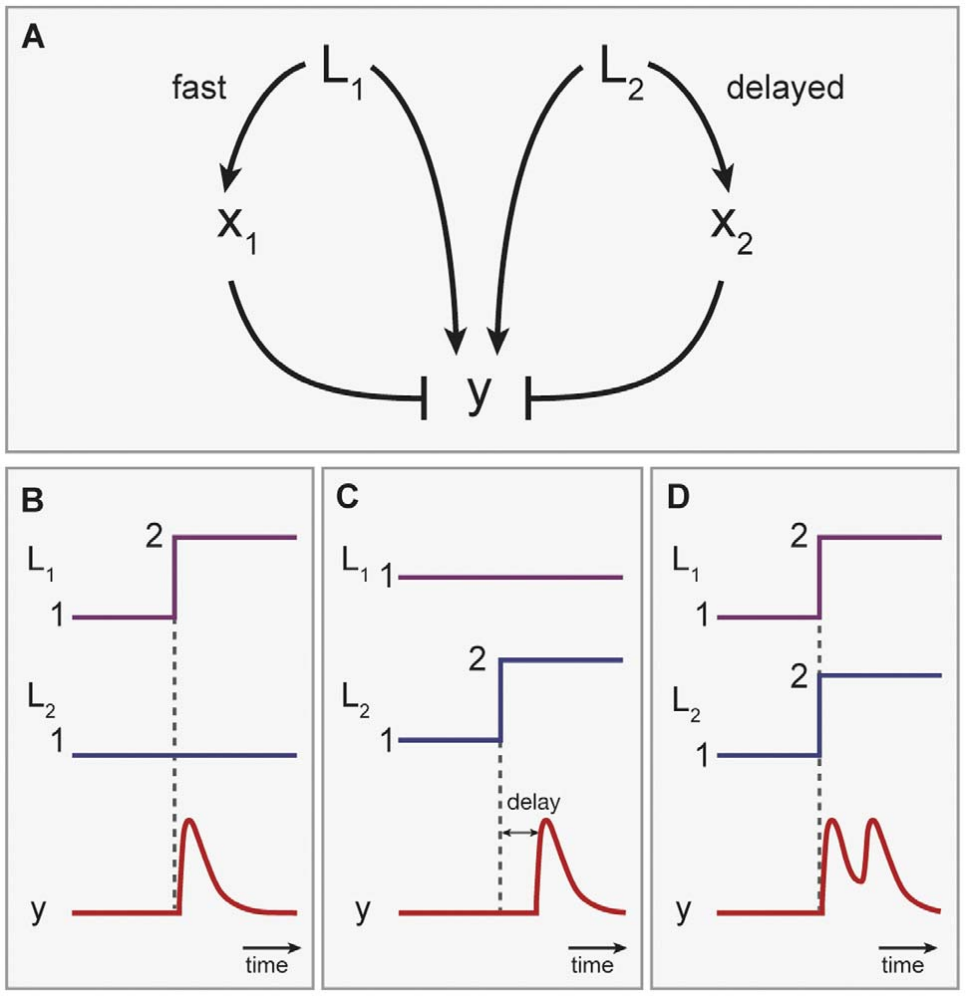
FCD systems with two internal layer components need not generally integrate multiple signals in a log-linear manner. (A) A model where each input interacts with its own internal layer component. In cases where one pathway has fast dynamics and the second pathway has a delay, the response to fold change steps in input one and two can be temporally separated. (B) Response to a step in L_1_ is a fast pulse. (C) Response to a step in L_2_ is a delayed pulse. (D) Response to simultaneous steps of L_1_ and L_2_ gives a two-pulse shape, and is thus not equivalent to any step of a single ligand.

Moreover, the separation of the response to the two inputs may allow a delayed response in one, such that the two inputs have separation of time scales as well. Consider the following system which has fold response to each of the signals
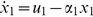
(30)

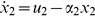
(31)

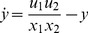
(32)although this system obeys the FCD conditions of Ref [9] for each signal on its own, it may not present an invariant dynamic response since 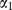
and 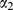 may dictate two different time scales, e.g. 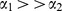, such that the system first reacts as if there is a change only in u_1_. Then, after the first dynamic pulse has ended, the second pulse initiates (see Fig.5). Note that in this case E_1_ cannot be defined: there is no stimulus on a single input that provides the same time-course as simultaneous stimuli of two inputs (see Fig. 5). Therefore, a separation of the internal layer components does not necessarily lead to an invariant dynamic response. However, such a system can in special cases still have simple integration rules for the two inputs, as shown in Material S1.

## Discussion

This study considered FCD sensory systems with multiple input signals. We show that when both signals impact the same receptor with equal number of binding sites, described by a MWC model [3], the integrated response is multiplicative: the system dynamics depend only on the product of the fold changes in the two stimuli F_1_F_2_. In cases where the inputs bind the same receptor with different number of sites, the result is a product of power laws, 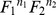. In both cases, plotting response amplitude or duration to step stimuli in the (log F_1_, log F_2_) plane is predicted to yield linear contours, with a slope 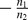, over the range of input concentrations in which FCD is valid (i.e. both ligand concentrations are in the range 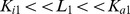 and 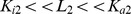). The cases of dependent ligand binding (interacting or exclusive) are discussed in Material S1, where we show that for a certain range of concentrations and parameters, interacting ligands can show FCD but exclusive binding of ligands excludes FCD. When the two inputs affect different receptors, or when the MWC picture is not applicable, other types of integration may be found and generally the system is not constrained to respond in a multiplicative (log-linear) way.

These results may address the question of how sensory systems compare apples and oranges. When the two signals sensed by the system are very different in nature (for example touch and noxious chemicals [7]), how can the system compare them? We find that FCD systems can compare different signals by relating each signal to its own previous level, by means of its fold-change, and then by multiplying these fold changes. In this way, each signal is normalized to its ambient level, enabling the comparison of two ‘non-dimensional’ factors, namely the fold-change in each signal.

By suitably designing the detector elements, the sensory system can create contours of equal response, with a given slope in the (logF_1_, logF_2_) plane, that appropriately weigh changes in the inputs to produce a desired response. Thus, a higher sensitivity to fold change in signal one than in signal two can be achieved by making the effective cooperativity coefficient n_1_ for signal one larger than for signal two.

One avenue for further research is to study more general models that provide FCD, and ask what is the largest class of signal integration functions spanned by all FCD mechanisms.

The present considerations may apply to a range of systems from molecular detection by receptors, neuronal sensory systems in simple organisms, and perhaps also to physiological sensory systems in humans such as taste and smell. A psychophysical experiment on taste, for example, may use different stimuli sensed by the same receptors such as cold temperature and menthol [32], or hot temperatures and capsaicin [33]. An interesting prediction is that no response is predicted upon specific ratios of increase in one signal together with a decrease in the other.

## Methods

### General Solution for FCD Systems with Two Inputs and a Single Internal Layer Component

We now describe the general formula for integration of two input signals sensed by a single shared internal component (e.g. a shared receptor). For simplicity we start with the one ligand case and then expand to the case of two ligands. Consider the following general transformation [11]

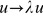
(33)

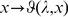
(34)


Then, following the procedure described in Eq.(14–22) one has [11].
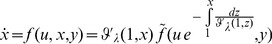
(35)

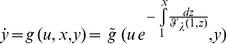
(36)


Note that since 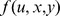
and 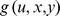
obey the FCD conditions of Shoval et al [9], 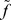 can be multiplied by a constant and maintain FCD response. Similarly, for two inputs and a general transformation: 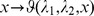 one has the following dynamic set of equations
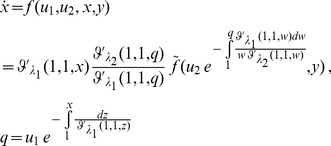
(37)

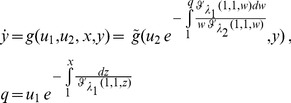
(38)


Eqn. (37–38) define the general formula for FCD systems which integrate two inputs by a shared single internal layer component.

## Supporting Information

Material S1 The online supplementary material includes additional information about the methods and results presented above. We present results for the cases when the two ligand bindings are dependent (interacting or exclusive). We also present special cases where a FCD system with two internal layer components has simple integration rules for the two inputs.
(DOCX)
