# Supplementary material for: Comparing Apples and Oranges: Fold-Change Detection of Multiple Simultaneous Inputs
Source: PLoS One. 2013 Mar 4;8(3):e57455. doi: 10.1371/journal.pone.0057455 (PMC3587607; doi:10.1371/journal.pone.0057455)
Supplement: Material S1 — The online supplementary material includes additional information about the methods and results presented above. We present results for the cases when the two ligand bindings are dependent (interacting or exclusive). We also present special cases where a FCD system with two internal layer components has simple integration rules for the two inputs. (DOCX) [file pone.0057455.s001.docx]

**Comparing apples and oranges: fold-change detection of multiple simultaneous inputs – Supporting Information Material S1**

Yuval Hart, Avraham E. Mayo, Oren Shoval and Uri Alon

Dept. Molecular Cell biology, Weizmann Institute of Science, Rehovot, Israel

Contents

[A MWC model for interacting ligands binding a shared receptor integrates signals in a log-linear manner 1](#_Toc316583976)

[A MWC model for exclusive binding of ligands to a shared receptor cannot hold FCD 3](#_Toc316583977)

[A system with two inputs, two internal layer components and an output with a FCD response for each input enables mapping of the two internal layer components output integration by studying the equivalent fold response to the two signal folds 3](#_Toc316583978)

# A MWC model for interacting ligands binding a shared receptor integrates signals in a log-linear manner

In the main text, we have assumed that binding of ligands to receptor is non-interacting. Here, we wish to relax this assumption and allow the two binding events to affect one another.

In order to analyze the case of interacting binding events, we use Tu et al. energy function formulation [1] of the MWC model [2]. In this formulation, the receptor has N_1_ subunits for binding ligand 1 and N_2_ subunits for binding ligand 2. Ligand occupancy of each subunit is denoted by $\sigma_{i_{1}}$ for ligand 1 and $\sigma_{i_{2}}$ for ligand 2 and can equal either zero or one: $\sigma_{i_{1}},\sigma_{i_{2}}=\{0,1\}$. In the concerted model, receptor activity s is either active (s=1) or inactive (s=0). Thus, the energy of the receptor depends on the variables s and $\sigma_{\{1,2\},i}$ as follows:

1. $H=s\left( H_{0}+\epsilon_{1}\Sigma_{i_{1}}\sigma_{i_{1}}+\epsilon_{2}\Sigma_{i_{2}}\sigma_{i_{2}}+\epsilon_{3}\left( \Sigma_{i_{1}}\sigma_{i_{1}} \right)\left( \Sigma_{i_{2}}\sigma_{i_{2}} \right) \right)+\mu_{1}\Sigma_{i}\sigma_{i_{1}}+\mu_{2}\Sigma_{i}\sigma_{i_{2}}+\mu_{3}\left( \Sigma_{i}\sigma_{i_{1}} \right)\left( \Sigma_{i}\sigma_{i_{2}} \right)$

Where $H_{0}$ is the energy difference between the active and inactive state in the absence of ligands. Each occupied receptor subunit suppresses receptor activity by increasing the energy of the active state by $\epsilon_{1}$ or $\epsilon_{2}$. The joint effect of both ligands occupation on the activity is described by the energy cost $\epsilon_{3}$. The energies $\mu_{1},\mu_{2}$ are the energies for ligand binding for the inactive state and depends on ligand concentrations and the dissociation constants of ligand to the inactive state, $K_{i,\{1,2\}}$. $\mu_{3}$ is the energy cost of having both ligand subunits occupied, and describes the interaction between ligand binding. The relation between energy parameters and the MWC model are:

1. $e^{-H_{0}}=K_{eq}, e^{-\epsilon_{1}}=C_{1}, e^{-\epsilon_{2}}=C_{2}, e^{-\epsilon_{3}}=C_{12}$
2. $e^{-\mu_{1}}=\frac{L_{1}}{K_{i,1}} ,e^{-\mu_{2}}=\frac{L_{2}}{K_{i,2}},e^{-\mu_{3}}=\frac{L_{1}L_{2}}{K_{i,12}}$

Where $K_{eq}$ is the equilibrium constant, L_1_ and L_2_ are the concentrations of ligand 1 and ligand 2 and the dissociation constants for the active state are given by$K_{a,\{1,2\}}=\frac{K_{i,\left\{ 1,2 \right\}}}{C_{\left\{ 1,2 \right\}}}$.

To calculate the average receptor activity, a= <s>, one needs to calculate the partition function $Z=\Sigma_{\{all states\}}e^{-H}$ and then differentiate its logarithm with respect to H_0_

1. $a=<s>=-\frac{1}{Z}\frac{\partial Z}{\partial H_{0}}=\frac{K_{eq}\left( 1+\frac{C_{1}L_{1}}{K_{i,1}} \right)^{N_{1}}\left( 1+\frac{C_{2}L_{2}}{K_{i,2}} \right)^{N_{2}}\left( 1+\frac{C_{12}L_{1}L_{2}}{K_{i,12}} \right)^{{N_{1}+N}_{2}}}{\left( 1+\frac{L_{1}}{K_{i,1}} \right)^{N_{1}}\left( 1+\frac{L_{2}}{K_{i,2}} \right)^{N_{2}}\left( 1+\frac{L_{1}L_{2}}{K_{i,12}} \right)^{{N_{1}+N}_{2}}+K_{eq}\left( 1+\frac{C_{1}L_{1}}{K_{i,1}} \right)^{N_{1}}\left( 1+\frac{C_{2}L_{2}}{K_{i,2}} \right)^{N_{2}}\left( 1+\frac{C_{12}L_{1}L_{2}}{K_{i,12}} \right)^{{N_{1}+N}_{2}}}$

Note that the case of no interactions between the two ligand binding events is achieved when $\epsilon_{3}=\mu_{3}=0$ which is equivalent to $C_{12}=1$ and $\frac{L_{1}L_{2}}{K_{i,12}}=1$ such that Eq.(S4) results in the known MWC formula for two ligands.

Generally, receptor activity does not follow a power-law behavior that will allow for a fold-change detection response. However, in the case where $K_{eq}$ is small compared with one, and ligand concentrations are within a range, such that they hold: $K_{i,12}\ll L_{1}L_{2}\ll\frac{K_{i,12}}{C_{12}}$ then power-law behavior is achieved for two cases: One, when ligands concentrations are within the range $K_{i,1}\ll L_{1}\ll\frac{K_{i,1}}{C_{1}}$ and $K_{i,2}\ll L_{2}\ll\frac{K_{i,2}}{C_{2}}$. In this case the receptor activity is given by:

1. $a=\frac{K_{eq}}{\left( \frac{L_{1}}{K_{i,1}} \right)^{N_{1}}\left( \frac{L_{2}}{K_{i,2}} \right)^{N_{2}}\left( \frac{L_{1}L_{2}}{K_{i,12}} \right)^{{N_{1}+N}_{2}}}$

In a sense, this case resembles the case of non-interacting binding because the above conditions are met when $K_{i,1}K_{i,2}\simeq K_{i,12}$ and $C_{1}C_{2}\simeq C_{12}$ which is met when two ligand binding is the multiplication of single binding probabilities.

The second case occurs when ligand concentrations are very low, meaning when $L_{1}\ll K_{i,1}$ and $L_{2}\ll K_{i,2}$ but yet $K_{i,12}\ll L_{1}L_{2}\ll\frac{K_{i,12}}{C_{12}}$ such that the receptor activity is given by:

1. $a=K_{eq}\frac{1}{\left( \frac{L_{1}L_{2}}{K_{i,12}} \right)^{{N_{1}+N}_{2}}}$

This condition is met when $K_{i,12}\ll K_{i,1}K_{i,2}$ which means the two ligands are synergistic in the sense that binding of the two ligands together is highly favorable compared to binding of each one of them by itself.

In both of these cases, the FCD demands are met and the log-linear multiplicative nature of the ligands is kept.

# A MWC model for exclusive binding of ligands to a shared receptor cannot hold FCD

For the case of exclusive binding, one can use the same Hamiltonian as used for the non-interacting ligands, but count only states which are exclusive, meaning that only ligand 1 or ligand 2 bind the receptor, but not together. The resulting receptor activity is given by:

1. $a=\frac{K_{eq}\left( \left( 1+\frac{C_{1}L_{1}}{K_{i,1}} \right)^{N_{1}}+\left( 1+\frac{C_{2}L_{2}}{K_{i,2}} \right)^{N_{2}} \right)}{\left( 1+\frac{L_{1}}{K_{i,1}} \right)^{N_{1}}+\left( 1+\frac{L_{2}}{K_{i,2}} \right)^{N_{2}}+K_{eq}\left( \left( 1+\frac{C_{1}L_{1}}{K_{i,1}} \right)^{N_{1}}+\left( 1+\frac{C_{2}L_{2}}{K_{i,2}} \right)^{N_{2}} \right)}$

Here, since the two ligands are summed rather than multiplied, there is no regime where the receptor activity can be represented by a power-law of both ligands. The only case for a possible FCD regime is the trivial case where one ligand concentration is extremely small and then FCD can be attained for the second ligand in its FCD range ($K_{i,1}\ll L_{1}\ll\frac{K_{i,1}}{C_{1}}$).

# A system with two inputs, two internal layer components and an output with a FCD response for each input enables mapping of the two internal layer components output integration by studying the equivalent fold response to the two signal folds

In this section we describe a specific setting which could yield a mapping between the internal components integration scheme and the fold response to the two signals: Consider a case where the two internal layer components have the same time scales and in addition the system shows exact adaptation in both internal layer components.

In this case, the two internal layer components, x_1_ and x_2_, have an explicit integral feedback form [3–5] on the output y. This means that one can write their dynamic equations in the following form

1. $\dot{x_{1}}=G_{1}\left( y-y_{0} \right)f_{1}(u_{1},x_{1})$
2. $\dot{x_{2}}=G_{2}\left( y-y_{0} \right)f_{2}(u_{2},x_{2})$

where $G_{1}\left( y-y_{0} \right)$ has a zero at $y_{0}$, the same zero as $G_{2}\left( y-y_{0} \right)$ in order for the system to reach the exact same stationary output level, $y_{0}$. Then, for y close to the steady-state value,$y_{0}$, the dynamic equations (S8-S9) have a conserved quantity, $\dot{x_{1}}f_{2}\left( u_{2},x_{2} \right)-\dot{x_{2}}f_{1}\left( u_{1},x_{1} \right)=0$. Thus, we get the following implicit equation:

1. $\dot{\frac{x_{1}}{f_{1}\left( u_{1},x_{1} \right)}}=\dot{\frac{x_{2}}{f_{2}\left( u_{2},x_{2} \right)}}$

In turn, this leads to a mapping of x_1_ onto x_2_,$x_{1}=Q\left( x_{2} \right)$ and so the two internal layer variables are coupled by the integral feedback mechanism.

The equivalent response of one ligand to a fold change in both ligands manifests the integration scheme of the internal layer components (x_1_,x_2_) to the output of the system (y). The dynamic equation of y holds: $\dot{y}=g\left( \frac{u_{1}}{x_{1}},\frac{u_{2}}{x_{2}},y \right)=g\left( \frac{u_{1}}{{Q(x}_{2})},\frac{u_{2}}{x_{2}},y \right)=g\left( \frac{u_{1}}{x_{1}},\frac{u_{2}}{{Q^{-1}(x}_{1})},y \right)$. Hence, one can map the 'input function' of the output y(t) by mapping the output's response to different folds.

Therefore, an FCD system with an explicit exact adaptation and no delays in both inputs can be internally mapped by studying the invariant response for different fold changes in the two ligands.

**References**

1. Mello BA, Tu Y (2005) An allosteric model for heterogeneous receptor complexes: Understanding bacterial chemotaxis responses to multiple stimuli. Proceedings of the National Academy of Sciences of the United States of America 102: 17354–17359. doi:10.1073/pnas.0506961102.

2. Monod J, Wyman J, Changeux J-P (1965) On the nature of allosteric transitions: A plausible model. Journal of Molecular Biology 12: 88–118. doi:10.1016/S0022-2836(65)80285-6.

3. Shoval O, Goentoro L, Hart Y, Mayo A, Sontag E, et al. (2010) Fold-change detection and scalar symmetry of sensory input fields. Proceedings of the National Academy of Sciences 107: 15995–16000. doi:10.1073/pnas.1002352107.

4. Barkai N, Leibler S (1997) Robustness in simple biochemical networks. Nature 387: 913–917.

5. Yi T-M, Huang Y, Simon MI, Doyle J (2000) Robust perfect adaptation in bacterial chemotaxis through integral feedback control. Proceedings of the National Academy of Sciences of the United States of America 97: 4649–4653.
